# The Modulation Transfer Function for Speech Intelligibility

**DOI:** 10.1371/journal.pcbi.1000302

**Published:** 2009-03-06

**Authors:** Taffeta M. Elliott, Frédéric E. Theunissen

**Affiliations:** 1 Helen Wills Neuroscience Institute, University of California Berkeley, Berkeley, California, United States of America; 2 Department of Psychology, University of California Berkeley, Berkeley, California, United States of America; University College London, United Kingdom

## Abstract

We systematically determined which spectrotemporal modulations in speech are necessary for comprehension by human listeners. Speech comprehension has been shown to be robust to spectral and temporal degradations, but the specific relevance of particular degradations is arguable due to the complexity of the joint spectral and temporal information in the speech signal. We applied a novel modulation filtering technique to recorded sentences to restrict acoustic information quantitatively and to obtain a joint spectrotemporal modulation transfer function for speech comprehension, the *speech MTF*. For American English, the speech MTF showed the criticality of low modulation frequencies in both time and frequency. Comprehension was significantly impaired when temporal modulations <12 Hz or spectral modulations <4 cycles/kHz were removed. More specifically, the MTF was bandpass in temporal modulations and low-pass in spectral modulations: temporal modulations from 1 to 7 Hz and spectral modulations <1 cycles/kHz were the most important. We evaluated the importance of spectrotemporal modulations for vocal gender identification and found a different region of interest: removing spectral modulations between 3 and 7 cycles/kHz significantly increases gender misidentifications of female speakers. The determination of the speech MTF furnishes an additional method for producing speech signals with reduced bandwidth but high intelligibility. Such compression could be used for audio applications such as file compression or noise removal and for clinical applications such as signal processing for cochlear implants.

## Author Summary

The sound signal of speech is rich in temporal and frequency patterns. These fluctuations of power in time and frequency are called modulations. Despite their acoustic complexity, spoken words remain intelligible after drastic degradations in either time or frequency. To fully understand the perception of speech and to be able to reduce speech to its most essential components, we need to completely characterize how modulations in amplitude and frequency contribute together to the comprehensibility of speech. Hallmark research distorted speech in either time or frequency but described the arbitrary manipulations in terms limited to one domain or the other, without quantifying the remaining and missing portions of the signal. Here, we use a novel sound filtering technique to systematically investigate the joint features in time and frequency that are crucial for understanding speech. Both the modulation-filtering approach and the resulting characterization of speech have the potential to change the way that speech is compressed in audio engineering and how it is processed in medical applications such as cochlear implants.

## Introduction

**Figure 1:**
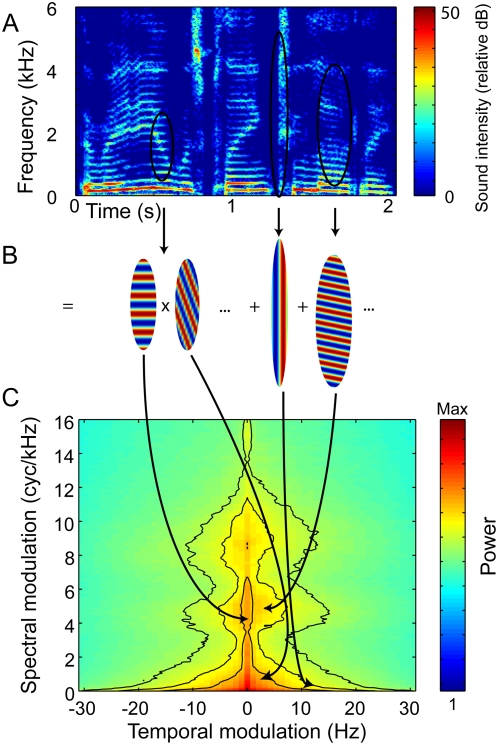
Component spectrotemporal modulations make up the modulation spectrum. (A) Spectrogram of a control condition sentence, “The radio was playing too loudly,” reveals the acoustic complexity of speech (Audio S1). All supporting sound files have been compressed as .mp3 files for the purpose of publication; original .wav files were used as stimuli. (B) Example spectrotemporal modulation patterns circled in the sentence (A) can be described as a time-varying weighted sum of component modulations. (C) The MPS shows the spectral and temporal modulation power in 100 sentences. The outer, middle, and inner black contour lines delineate the modulations contained in 95%, 90%, and 85% of the modulation power, respectively. Down-sweeps in frequency appear in the right quadrant, whereas upward drifts in frequency are in the left quadrant. Slower temporal changes lie near zero on the axis, while faster changes result in higher temporal modulations towards the left and right of the graph.

Human speech, like most animal vocalizations, is a complex signal whose amplitude envelope fluctuates timbrally in frequency and rhythmically in time. Horizontal cross-sections of the speech spectrogram as in Figure 1A describe the time-varying envelope for a particular frequency while vertical cross-sections at various time points show spectral contrasts, or variation in the spectral envelope shape (Audio S1). Indeed, the structure in the spectrogram of speech is not characterized by isolated spectrotemporal events but instead by sinusoidal patterns that extend in time and frequency over larger time windows and many frequency bands. It is well known that it is these patterns that carry important phonological information, such as syllable boundaries in the time domain, formant and pitch information in the spectral domain, and formant transitions in the spectrotemporal domain as a whole [1]. In order to quantify the power in these temporal and spectral modulations, the two-dimensional (2D) Fourier transform of the spectrogram can be analyzed to obtain the modulation power spectrum (MPS) of speech [2],[3]. In this study, first we repeated this analysis using a time-frequency representation that emphasized differences in formant structure and pitch structure. Then we used a novel filtering method to investigate which spectral and temporal modulation frequencies were the most important for speech intelligibility. In this manner we obtained the speech modulation transfer function (speech MTF). We were then able to compare the speech MTF with the speech MPS in order to interpret the effect of modulation filters on perception of linguistic features of speech.

Our study both complements and unifies previous speech perception experiments that have shown speech intelligibility to depend on both spectral and temporal modulation cues, but to be surprisingly robust to significant spectral or temporal degradations. Speech can be understood with either very coarse spectral information [4]–[8] or very coarse temporal information [9]–[11]. Our goal was to unify spectral and temporal degradation experiments by performing both types of manipulations in the same space, namely, the space of joint spectrotemporal modulations given by the speech MPS. The approach makes advances in the rigor of signal processing, in the specificity of the manipulations allowed, and in the comparison with speech signal statistics. First, the approach depicts visually and quantifies the concomitant effects that temporal manipulations have on the spectral structure of the signal, and that spectral filtering has on temporal structure. Second, the technique offers the possibility of notch filtering in the spectral modulation domain, something which has not been done before. Whereas degradation by low-pass filtering can reveal the minimum spectral or temporal resolution required for comprehension, notch filtering can distinguish more limited regions of spectrotemporal modulations that differ in levels of importance for comprehension. Third, the modulation filtering technique can be used to target specific joint spectral and temporal modulations. In this study, this advantage was exploited in a two-step filtering procedure to measure the effects of precise temporal and spectral degradations in the range of modulations most important for intelligibility. In this procedure, we first removed potentially redundant information in higher spectral *and* temporal modulations, and then we applied notch spectral or temporal filters within the remaining modulation space. Finally, we were able to compare the results of the speech filtering experiments to the MPS of speech, in order to make an initial characterization of the speech MTF in humans. As far as we know, this is the first such comparison using a linear frequency axis and a modulation transfer function obtained directly from speech intelligibility experiments. The resultant speech MTF could be used to design more optimal speech compression such as that required by cochlear implants.

Neurophysiological research on animal perception of modulations inspired our study. While the cochlea and peripheral auditory neurons represent acoustic signals in a time-frequency decomposition (a cochleogram), higher auditory neurons acquire novel response properties that are best described by tuning sensitivity to temporal amplitude modulations and spectral amplitude modulations (reviewed in [12] and [13]). By designing human psychological experiments using the same representations used in neurophysiological research, we can begin to link brain mechanisms and human perception.

Speech signals carry information about a speaker's emotion and identity in addition to the message content. As a final thrust of investigation, we tested whether modulations corresponding to acoustic features embedded in the speech signal enabled listeners to detect the gender of the speaker. Vocal gender identity has been shown to depend on some spectral features in common with, and some distinct from, the spectral features conferring speech intelligibility [14],[15].

## Results

Spectrotemporal modulations underlying speech intelligibility and gender recognition were assessed in psychophysical experiments using sentences in which spectrotemporal modulations had been systematically filtered. Since our psychophysical experiments were in large part inspired by our analysis of the spectrotemporal modulations of speech, we begin by reporting the resulting modulation space. We will describe the characteristics of the MPS of speech and emphasize which characteristics are general to natural sounds, which are general to animal vocal communication signals, and which ones are more unique to human speech. The goal of the psychophysical experiments was to determine the subset of perceptible modulations that contribute exceptionally to speech intelligibility.

### Modulation Power Spectrum of Speech

The MPS of American English (Figure 1C) was calculated from a corpus of 100 sentences (see Materials and Methods). This speech modulation spectrum shares key features observed in other natural sounds. As in all natural sounds, most of the power is found for low modulation frequencies and decays along the modulation axes following a power law [3]. Moreover, as typical of animal vocalizations, the MPS is not separable; most of the energy in high spectral modulations occurs only at low temporal modulation, and most high temporal modulation power is found at low spectral modulation [3],[16]. This characteristic non-separability of the MPS is due to the fact that animal vocalizations contain two kinds of sounds: short sounds with little spectral structure but fast temporal changes (contributing power along the x-axis at intermediate to high temporal frequencies), and slow sounds with rich spectral structure (found along the y-axis at intermediate to high spectral frequencies). In normal speech, this grouping of sounds corresponds roughly to the vocalic (slow sounds with spectral structure, produced with phonation) and non-vocalic acoustic contrasts (fast sounds with less spectral structure, produced without phonation). Animal vocalizations and human speech do have sound elements at intermediate spectrotemporal modulations, but these have less power (or in other words are less frequent) than expected from the power (or average occurrence) of spectral or temporal modulations taken separately, reflecting the non-separability of the MPS.

**Figure 2:**
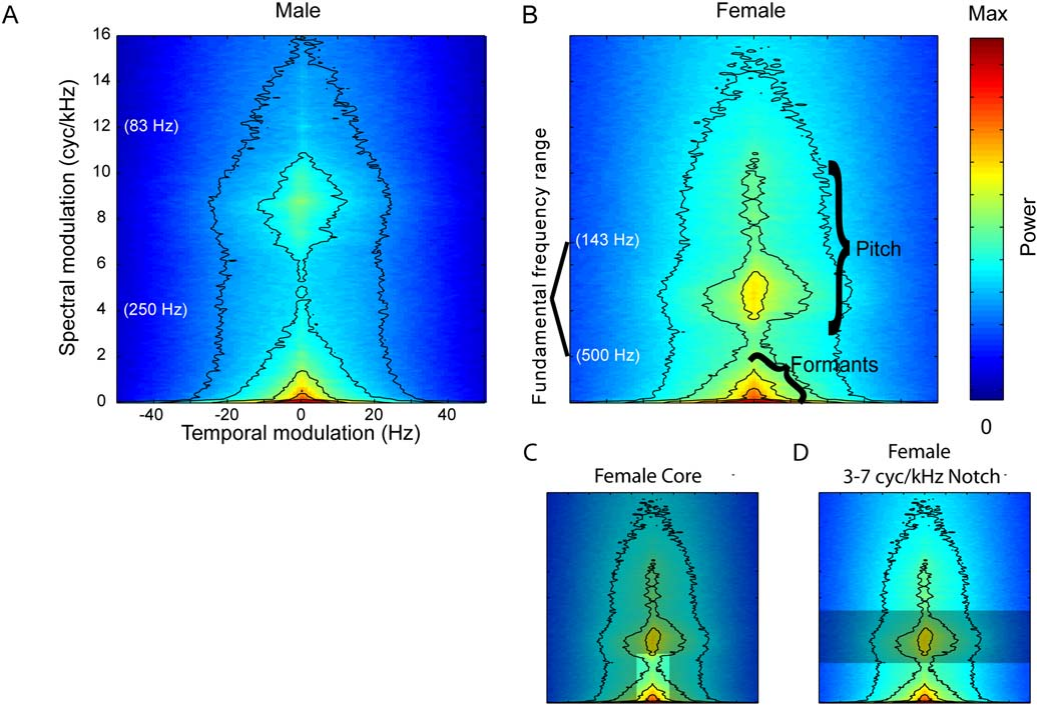
Spectral modulations differ in male and female speech. (A,B) The MPS of the 50 corpus sentences spoken by males (A), and of the 50 spoken by females (B), with black contour lines as in Figure 1. White parenthetical labels on the y-axes of (A) and (B) show related frequencies demarcating the male and female vocal registers; they correspond to spectral modulations based on harmonic spacing. (C,D) Modulation filters that resulted in misidentification of the speaker's gender. (C) the speech MPS for female speakers is overlapped with the boundaries of the low-pass spectrotemporal filter. In this condition, speaker gender was misidentified in a quarter of the sentences, with 91% of those errors being females misidentified as male. (D) the same female speech MPS overlapped with a notch filter that removed modulations from 3 to 7 cycles/kHz. Of the 21% gender errors in this condition, 95% were female speakers misidentified as male.

An additional aspect of human speech is that modulations separate into three independent areas of energy along the axis of spectral modulation, at low temporal modulation (Figures 1C and 2). First, the triangular energy area at the lower spectral modulation frequencies corresponds to the coarse spectral amplitude fluctuations imposed by the upper vocal tract, namely the formants and formant transitions (labeled in Figure 2B). The other two areas of spectral modulation energy, found at higher levels, correspond to the harmonic structure of vocalic phones produced by the glottal pulse; this energy diverges into two areas because of the difference in pitch between the low male voice (highest spectral modulations) and the higher female voice (more intermediate spectral modulations). The lower register of the male voice produces higher spectral modulations because of the finer spacing of harmonics over that low fundamental. Equivalent pitches corresponding to the spectral modulations are labeled parenthetically in white on the y-axis of Figure 2. The MPS can also be estimated from time-frequency representations that have a logarithmic frequency axis (see Materials and Methods, and Figure S1). Although log-frequency representations are better models of the auditory periphery, the linear-frequency representation is more useful for describing the harmonic structure present in sounds. For example, the separation of the spectral structure of vocalic phones into three regions is a property that is observed only in the linear frequency representation (Figure S1).

Thus, in the speech MPS with linear frequency, not only do vocalic and non-vocalic sounds occupy different regions within the modulation space, but the spectral modulations for vocalic sounds corresponding to formants and male and female pitch occupy distinct regions. Also, human speech is symmetric between positive and negative temporal modulation frequencies, showing that there is equal power for upward frequency modulations (Figure 1C, left quadrant) and downward frequency modulations (right quadrant).

### Psychophysical Experiments in Spectrotemporal Modulation Filtering

Our modulation filtering methodology allowed us not only to rigorously degrade speech within its spectral and temporal structure but also to relate the results from the degradation to acoustic features of the signal that are important for different percepts, as described above. Our psychophysical experiments are organized in three sections. We first report results from the two sets of modulation filters applied to the whole spectrotemporal modulation spectrum of speech—low-pass filters and notch filters—which indicated a subset of modulations that are critical for speech understanding, thereafter designated the “core” modulations. Subsequently, we report results from notch filters applied to sentences containing only core modulations, further refining our identification of crucial spectrotemporal modulations.

#### Low-pass modulation filtering

**Figure 3:**
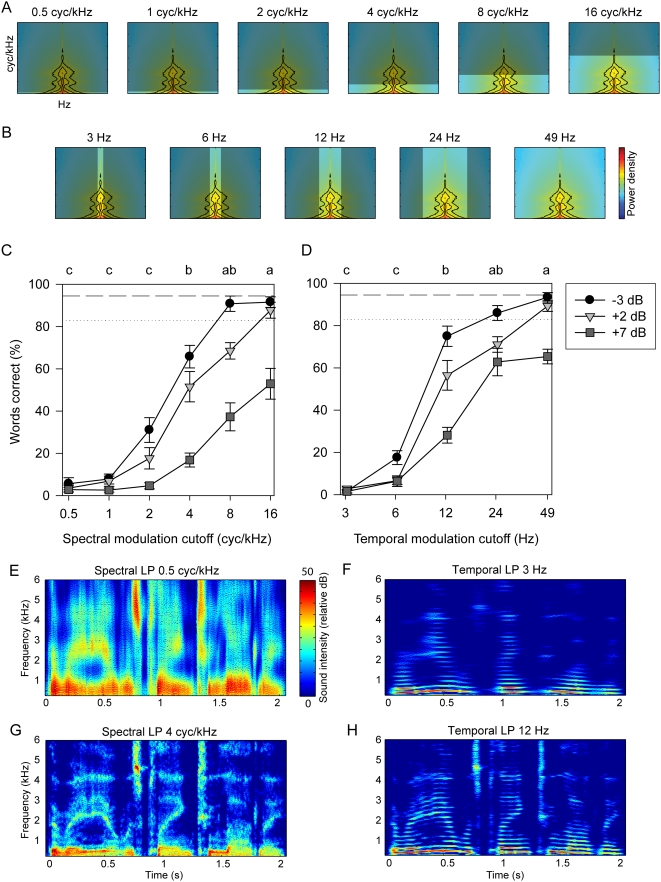
Comprehension of low-pass modulation filtered sentences. (A,B) Grayed areas of thumbnails show spectrotemporal modulations removed by low-pass modulation filtering in the spectral (A) or temporal (B) domain. Units and axis ranges are the same as in Figure 2. Each thumbnail represents a stimulus set analyzed in (C,D). (C,D) Mean±s.e. performance in transcribing words from the low-pass modulation filtered sentences. Cutoff frequencies on the x-axes of the two graphs are presented in units appropriate to the spectral or temporal domain, but could equally well be viewed on one continuous scale in either unit. Symbols show SNR levels. Dashed line shows control performance at +2 dB SNR; dotted line shows control performance at −3 dB SNR. Points at cutoff frequencies which share no capital letters in common (above line plots) are significantly different (repeated measures ANOVA, Bonferroni post-hoc correction, p<0.0008) at the +2 dB SNR condition. (E and G) Spectrograms of an example sentence (same as in Figure 1) with the most extreme spectral modulation filtering (with a low-pass cutoff of 0.5 cycles/kHz; Audio S2) and the spectral modulation filtering at which comprehension became significantly worse (4 cycles/kHz; Audio S3), respectively. LP = Low-pass. (F and H) Spectrograms of the example sentence with the most extreme temporal modulation filtering tested (having a low-pass cutoff of ∼3 Hz; Audio S4), and the temporal modulation filtering at which comprehension became significantly worse (cutoff 12 Hz; Audio S5).

We scored the number of words reported correctly from sentences with low-pass filtered spectral or temporal modulations (see Materials and Methods for the modulation filtering procedure) at cutoff frequencies roughly logarithmically distributed across the speech MPS (Figure 3). Sentences were embedded in noise and played back at 3 different levels of signal-to-noise ratio (SNR). Comprehension dropped off significantly at around 4 cycles/kHz low-pass cutoff spectral frequency, and at 12 Hz in the temporal domain, with a further significant decrease at 6 Hz. Gray shading in the thumbnails of the modulation spectrum show the modulations of speech that were low-pass filtered spectrally (Figure 3A), or temporally (Figure 3B). The line graphs (Figure 3C and 3D) show mean±s.e. performance on the sentence comprehension test for the spectral and the temporal conditions, at the three SNRs. Spectrograms of the example sentence from Figure 1 show extreme spectral (0.5 cycles/kHz, Figure 3E, Audio S2) and temporal smearing (3 Hz, Figure 3F, Audio S3), in addition to the spectral smearing (4 cycles/kHz, Figure 3G, Audio S4) and temporal smearing (12 Hz, Figure 3H, Audio S5) conditions at which comprehension decreased significantly in comparison to control.

Together, the results from the spectral and temporal domains suggested that there exists a region, or “core”, of modulations below ∼4 cycles/kHz and ∼8 Hz that are essential for comprehension. Sentences containing only these core modulations served afterwards as a control condition and as starting material for further notch filtering. In a separate experiment, we also applied low-pass spectral filtering using spectral modulations obtained from a logarithmic frequency axis in the time-frequency representation (see Figure S1). Those data show that spectral modulations below 2 cycles/octave are important (for a center frequency of 500 Hz, 2 cycles/octave = 4 cycles/kHz). Finally, we also examined the effect of low-pass modulation filtering on nuclear vowel (h/V/d) and consonant discrimination (/C/a). Vowels were less affected by temporal filtering and consonants were less affected by spectral filtering (data not shown).

#### Notch modulation filtering

Next, we tested the effect of notch filters on speech comprehension. The widths of the notch filters were designed to be logarithmically proportional because the modulation power in the signal decreases following a power law from the origin in both the spectral and temporal dimensions [3]. Also, psychophysical experiments suggest that the Q factor of the human temporal modulation filter is constant for frequencies up to 64 Hz [17], and comparative judgments of auditory duration follow Weber-Fechner's law [18],[19]. All notch filtering experiments were performed at the intermediate SNR level of +2 dB.

**Figure 4:**
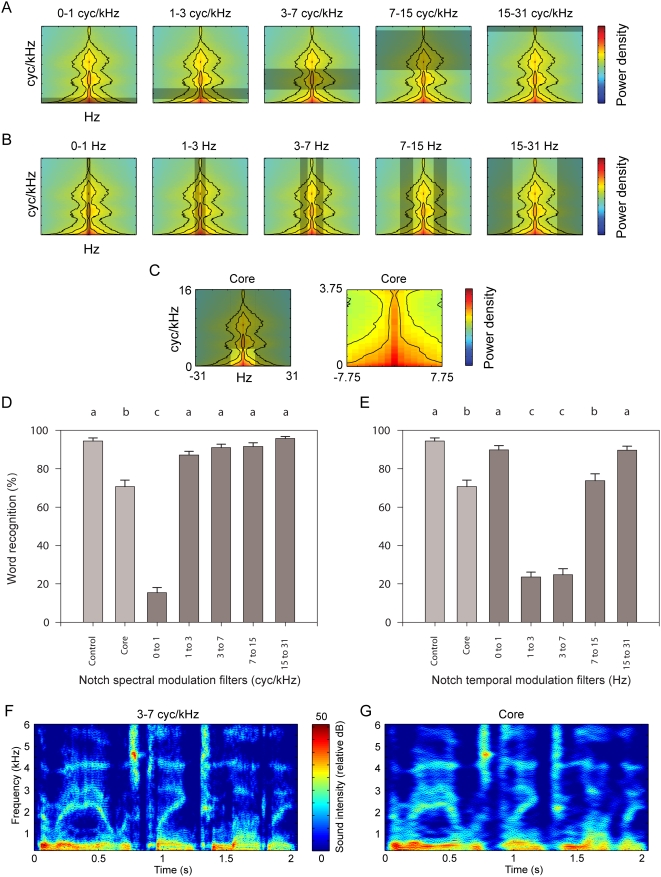
Comprehension of speech with notch-filtered modulations or “core” modulations. (A–C) The speech modulation spectrum with filtered modulations denoted by grayed areas as in Figure 5A. (A) Spectral notch modulation filters. (B) Temporal notch modulation filters. (C) Core modulations most essential to comprehension in Figure 5 are depicted in full and zoomed-in thumbnail plots. Stimuli for the core condition were obtained by low-pass filtering in both the spectral and temporal modulation domains. (D,E) Mean±s.e. comprehension when either spectral (D) or temporal (E) modulation filters were applied to the sentences, along with control sentences (lighter gray bars) containing all or only core modulations (C). Stimulus conditions which share no lower case letters (above plots) in common are significantly different, as in Figure 5 (repeated measures ANOVA). (F) Spectrogram of the example sentence after spectral modulations between 3 and 7 cycles/kHz were filtered out (Audio S6). (G) Spectrogram of the example sentence containing only the core of essential modulations below 7.75 Hz and 3.75 cycles/kHz (Audio S7).

As in Figure 3, thumbnails of the modulation spectrum in Figure 4 depict filtered areas layered in transparent gray. Note that temporal notch filters removed both positive and negative modulations, appearing as symmetric grayed areas (Figure 4B). Bar graphs (Figure 4D and 4E) show average±s.e. word comprehension. Light gray bars in these graphs denote the control condition (spectrogram inversion without modulation filtering) and the core condition in which inessential modulations were removed, by first low-pass filtering at 3.75 cycles/kHz and then at 7.75 Hz (Figure 4C and 4G). Dark gray bars in the graphs show the performance for each of the five spectral (Figure 4A; see one example in Figure 4F, Audio S6) and five temporal notch modulation filters (Figure 4B).

Comprehension of core modulations was 75% word recognition (example sentence spectrogram in Figure 4G, Audio S7). Of the spectrally delimited filtering, only the removal of modulations below 1 cycles/kHz significantly decreased sentence comprehension relative to control performance (Figure 4D). In the temporal domain, the 7–15 Hz notch filter caused a small but significant decrease in intelligibility, yielding performance that was at a level similar to the core condition (Figure 4E). More importantly, the removal of intermediate temporal modulations (either from 1–3 Hz or from 3–7 Hz) produced a significantly greater decrement in performance (Figure 4E and 4F).

#### Notch filtering of the “core” modulations

Since the initial low-pass filtering experiment had revealed that spectral modulations below ∼4 cycles/kHz and temporal modulations below ∼8 Hz are essential for comprehension, we limited modulations to this core spectrotemporal range (<3.75 cycles/kHz and <7.75 Hz) and further applied notch filters to test which core modulations contribute most to comprehension. This dual filtering allowed us to remove potentially redundant information found at modulations outside of the core.

**Figure 5:**
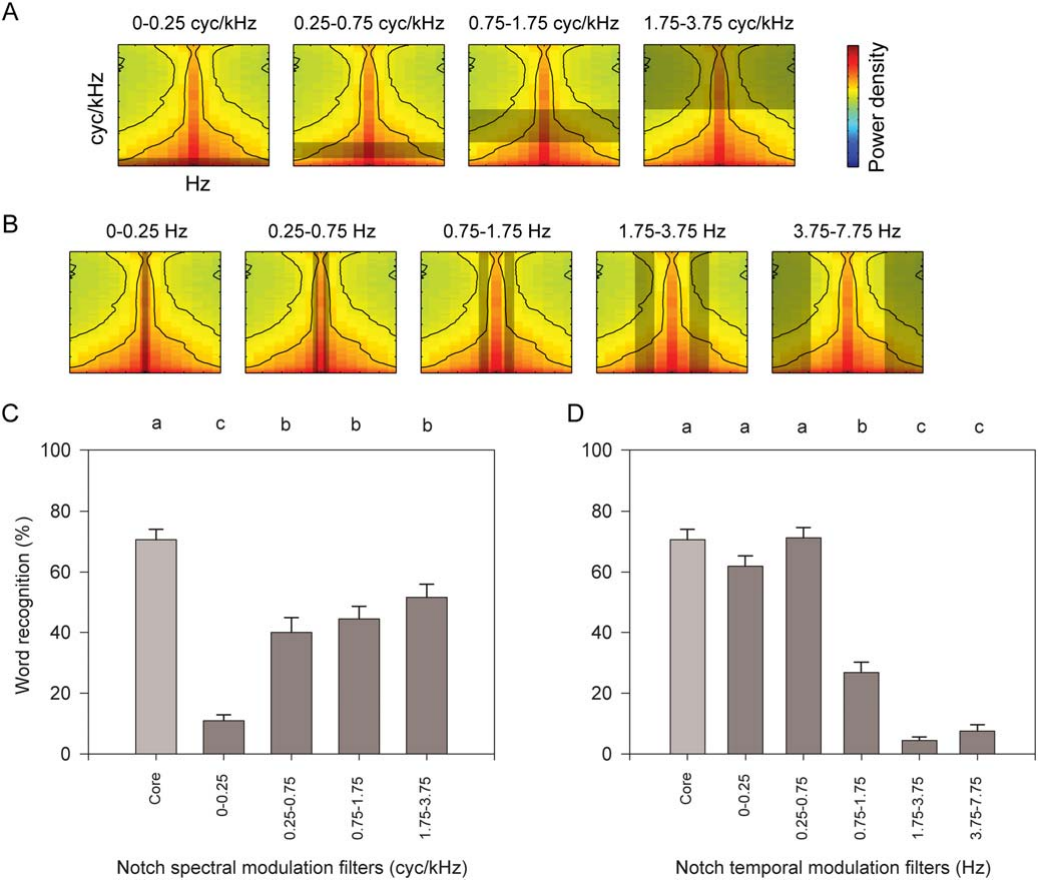
Comprehension of “core” modulations in speech with notch filtering. (A,B) Notch filters in the spectral (A) or temporal (B) modulation domain removed modulations from sentences that contained only core modulations after having been low-pass filtered in both domains. As depicted in Figure 4C, x- and y-axes are 0 to ±7.75 Hz and 0 to 3.75 cycles/kHz, respectively. (C,D) Comprehension when spectral (C) or temporal (D) notch filters were applied to sentences containing only core modulations. See Figure 6C for a thumbnail of the core modulations. As in Figures 5 and 6, conditions which share no lower-case labels in common are significantly different (repeated measures ANOVA).

Figure 4C shows the core of modulations (right thumbnail is a zoom-in of left thumbnail; the magnified scale is used in the thumbnails of Figure 5A and 5B). Sentences limited to the core modulations provided the control condition since in this experiment the notch filters were applied to them (Figure 5A and 5B) instead of to sentences without any of the perceptible modulation spectrum previously removed (as in the other experiments; Figure 3A and 3B). As explained above, the grayed areas in the thumbnail modulation spectra (Figure 5A and 5B) show which modulations were removed in each condition. Notch boundaries were again logarithmically spaced. There were only four spectral notches because the core modulations are already more limited in the spectral than the temporal domain.

Notch filters removing any of the core spectral modulations resulted in a decrease in intelligibility but this was especially true for the notch at the lowest modulation frequency (below 0.25 cycles/kHz) (Figure 5C). In the temporal dimension, any of the three temporal modulation notch filters above 0.75 Hz resulted in a decrease in performance, but in this case the effect was greater for higher temporal modulations (above 1.75 Hz), significantly decreased comprehension (Figure 5D).

The results of the notch filtering experiments show firstly that intermediate temporal modulations between 1 and 7 Hz are critical for speech intelligibility, whereas lower or higher temporal modulations are less critical. Secondly, the very low spectral modulations that we tested appeared to be critical. The human speech intelligibility transfer function appears therefore to show a band-pass tuning in the temporal domain and a low-pass tuning in the spectral domain.

### Gender Identification

Subjects reported the gender of the speakers of the notch-filtered sentences. Even though sentences having modulations restricted to the “core” (Figures 4D and 5C) were well comprehended, gender identification of the speakers of these sentences fell to 77%, where chance would be 50%. Of the gender errors, 91% occurred when the speaker was female. When modulations outside the core were spared, the notch-filter of spectral modulations between 3 and 7 cycles/kHz (Figures 2C and 4F) significantly decreased gender identification (to 79%). Of these misidentified speakers, 95% were female. Both the core condition and this spectral notch condition lacked modulations in the 3–7 cycles/kHz range, where female speech has more power (core spectral modulations are below 3.75 cycles/kHz). Male speech has more power shifted to higher spectral modulations (6–11 cycles/kHz). Thus, spectral modulation filters in the uniquely male range produced no significant decrease in gender identification. These results can be explained by the fact that whenever the filtered sentences lacked spectral modulation information unique to the female vocal register, subjects guessed that the speaker was male.

## Discussion

This study attempts to use dynamic properties of sound, rather than the traditionally stereotyped cues of acoustic phonetics, to refashion a parsimonious account of speech perception. Specifically, we used a novel filtering technique to remove spectrotemporal modulations from spoken sentences in order to isolate the acoustic properties critical for identifying linguistic features and for recognizing gender as a personal attribute of the voice.

We first systematically degraded sentences by filtering specific temporal and spectral modulation frequencies and then examined the effect on the number of words comprehended. As we will discuss in more detail below, our study replicates, but also has several advantages over, previous degradations performed in the temporal or spectral domain alone. First, it provides a rigorous mathematical framework to precisely quantify what is being removed from the speech signal. Second, the framework unifies manipulations across two lines of research that are otherwise described orthogonally, namely AM and FM filtering [20]. Finally, we can make a direct connection between the acoustical space we manipulated and the information-bearing features of speech distributed in each particular region [21].

In the MPS of American English (our prototype for human speech), the distinctive non-separable distribution of energy—namely, close to the x and y axes—corresponds roughly to a division between vocalic and non-vocalic sounds [3],[16],[21]. Non-vocalic phones in speech tend to be rapid and to have little spectral structure whereas vocalic phones are longer and have more spectral structure. Our categorization of speech sounds along the spectral and temporal axes of the MPS remains, of course, rather coarse. For example, voicing is associated with multiple acoustic properties and is only one of the linguistic features (e.g., place of articulation, manner, rounding) needed in order to categorize phonemes [22]. A more detailed analysis of the MPS of individual phonemes or simple combinations of phonemes would further illustrate the usefulness of this methodology for speech analysis [21].

Within the spectral structure especially associated with vocalic sounds, we also found a clear separation between pitch information and phonetic information (formants and formant transitions). The separation of the formant spectral frequencies from the pitch spectral frequencies had been described before and is one reason that cepstral analysis works well for the determination of formant frequencies [23]. In the discussion that follows, we will relate performance on the comprehension task to the acoustic features of speech we filtered from the MPS.

Our low-pass spectral-modulation filtering experiment shows that speech intelligibility begins to degrade significantly when modulations below 4 cycles/kHz are removed. Not surprisingly, this definitive point corresponds to the upper extent of the area in the speech MPS occupied by energy associated with formants (Figures 1 and 2). The separation between formant peaks in English vowels is greater than 500 Hz (or 2 cycles/kHz) [24], but finer spectral resolution (up to 4 cycles/kHz) would be beneficial to capture further the overall spectral shape of the formant filters and to detect formant transitions.

There is a large literature on how spectral degradation affects speech comprehension, the most similar studies being those of Shannon and Dorman and colleagues [5],[6] who have investigated speech intelligibility with very limited spectral resolution as one would experience with a cochlear implant. Shannon et al. [6] reported that speech intelligibility in a noise-free setting was fully preserved with spectral structure present in only 4 frequency channels below 4 kHz. These spectral bands would correspond in our implementation to a low-pass filter cutoff of approximately 1 cycles/kHz, or 1.7 cycles/octave, which is below the level needed for fully resolving formant spectral peaks and considerably below our cutoff of 4 cycles/kHz (or 2 cycles/octave as shown in Figure S1). However, when noise is present, Friesen et al. have shown that intelligibility increased with additional spectral channels [25]. In that study, for 0 dB SNR, 16 channels spaced below 6 kHz (or approximately 3.75 cycles/kHz) yielded significant additional comprehension over that of more degraded speech with fewer frequency channels. Our results are consistent with that result, and our study brings several additional insights to this analysis. First, the notch filtering experiments unequivocally demonstrate that the spectral MTF is truly low-pass. Removing lower (or intermediate) spectral modulations while preserving higher spectral modulations results in significant decreases in speech intelligibility. In other words, there does not appear to be information that is redundant between the spectral modulations below 4 cycles/kHz and higher spectral modulations (further details of the notch filtering results are discussed below). Second, our comparison between the speech MTF and the speech MPS offers an obvious explanation for the critical spectral frequency cutoff: it corresponds to the modulation power boundary of formants and formant transitions. Finally, by examining how much modulation power was removed in the filtering operations, we can also say that the crucial modulation areas are not simply the ones with the higher power in the speech MPS. For example, the region of the core notch filter between 0.25 and 0.75 cycles/kHz contributes less to intelligibility than the 0–0.25 cycles/kHz area, although the former contains higher power. Humans appear to be particularly adept at detecting very low modulations in the spectral envelope and at using that information for speech intelligibility.

In the temporal dimension, we showed that filtering the amplitude envelope of the speech signal below 12 Hz results in significant intelligibility deficits. Our results are similar to experiments in which the temporal envelope of speech was low-pass filtered or degraded. For Dutch, English and Japanese, it was shown that the region below 8 Hz is critical for speech comprehension [9]–[11]. This critical modulation is somewhere between the temporal modulations corresponding to the rate of syllables, at around 2 to 5 Hz [22], and those corresponding to phonemes, at around 15–30 Hz [26]–[28]. In the MPS, we observe that frequencies below 10 Hz account for approximately 85% of all the spectrotemporal modulation power. Examining the temporal modulation spectrum as a function of frequency bands (rather than as a function of spectral modulations, as shown in the MPS), Greenberg and Arai showed that the peak in power lies between 4 and 6 Hz [29]. By preserving frequencies below 8 Hz, one therefore retains most of the power in the temporal modulation spectrum. Qualitatively, the speech sounds that were heavily temporally filtered (below 5 Hz) sounded like reverberated speech, consistent with the observation that it is the higher temporal modulation frequencies that are affected in reverberant environments [30].

Interpreting which modulations proved crucial in the low-pass spectral or temporal filtering results is problematic because each relative lowering of the cutoff frequency removed increasingly more modulations. Furthermore, comparisons between low-pass cutoffs do not exclude the possibility that higher intelligibility could be achieved with isolated regions of the MPS. To obtain something akin to a modulation transfer function (MTF) for speech intelligibility, low-pass filtering manipulation must be complemented with high-pass filtering. Alternatively, a transfer function can be obtained directly from notch filtering experiments. We chose the latter approach and based the design of our notch filters on the results from the low-pass experiments. The combination of notch filtering and low-pass filtering also allowed us to examine areas in the speech MPS that carry redundant information.

Two conclusions can be made from the notch filtering experiments. First, the results show a low-pass spectral tuning with most of the gain between 0 and 1 cycles/kHz, and a band-pass temporal tuning with most of the gain between 1 and 7 Hz. Second, the results show the high level of redundancy in the speech signal. The intelligibility of most notch-filtered stimuli remained excellent. This is even more remarkable considering that tests were done with a SNR of 2 dB. Redundancy is evident also when one examines the difference in results obtained from the low-pass and notch filters. Notably, the low-pass cutoff spectral frequency of 2 cycles/kHz significantly reduced performance as compared to the 4 cycles/kHz condition, whereas the 1–3 and 3–7 cycles/kHz notch filters straddling that range of modulations produced no significant decrease in performance. This discrepancy suggests that some of the information about formant structure in the 1–4 cycles/kHz range can also be found at higher spectral modulation frequencies. For this reason, we conducted the second notch filter experiment that operated on the core modulations (modulations below ∼4 cycles/kHz and ∼8 Hz). This second notch experiment allowed us to obtain a more detailed MTF.

**Figure 6:**
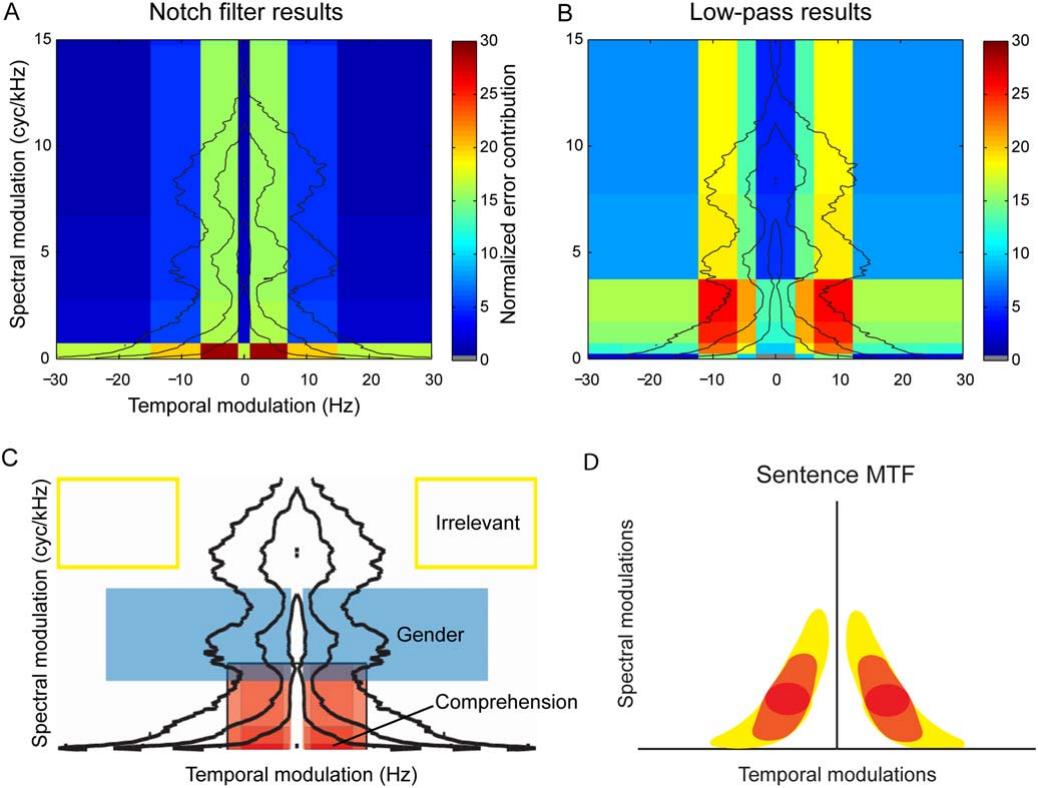
Combination of results from low-pass and notch modulation filtering. (A,B) To combine the spectral and temporal results from low-pass (A) and notch (B) modulation filtering, we calculated the average percent error in word comprehension, and divided by the average control comprehension. Then we multiplied the normalized errors from the spectral and temporal notch filters. Black lines indicate the contours of modulation power, as in Figure 1. Red areas are more important for speech comprehension than blue. The summary plot of the low-pass spectral and temporal modulation filters used the additional error caused by each subsequent lowering of the cutoff. Notch and low-pass experiments had somewhat different results in the spectral domain. The notch filtering implicated modulations closer to the origin, but still intermediate in temporal modulation, as most crucial. This discrepancy suggests a non-linearity in the relative contribution of modulations: the removal of intermediate spectral modulations matters more when higher spectral modulations are missing as well. (Dropping the low-pass cutoff spectral frequency from 4 to 2 cycles/kHz significantly reduced performance, but the 1–3 and 3–7 cycles/kHz notch filters straddling that range produced no significant difference.) (C) Schematic of modulations underlying comprehension and gender identification. The summary cartoon shows a region of low spectral and intermediate temporal modulations is of the greatest importance for speech intelligibility (red), while a separate band of higher spectral modulations (blue) make a speaker sound female. Yellow outlines the modulations that did not significantly contribute to sentence comprehension in any experiment. (D) Sentence modulation transfer function. When compression design, speech recognition by machines, and cochlear implant applications impose constraints on the bandwidth of a speech signal, modulation filtering could reduce a speech signal to only the modulation components needed for comprehension (red area). Depending on the bandwidth permitted, increasingly more of the orange and then yellow areas of the modulation spectrum could be included to add to the perception of vocal source characteristics.

The final speech MTF was obtained by combining the results of the spectral and temporal notch filters applied to the whole MPS. For this purpose, we calculated the average percentage error in word comprehension, and divided by the average control comprehension. Then we multiplied the normalized comprehension errors from the spectral notch filters (Figure 6A, vertical stripes), and the temporal notch filters (Figure 6A, horizontal stripes). The resulting color plot indicates which MPS areas are more important (red) for speech comprehension, and which are less important (blue). For comparison, we also generated a summary plot from the low-pass spectral and temporal modulation filters (Figure 6B). In this case, the subsequent increases in error caused by each lowering of the cutoff modulation frequency were used. A similar analysis of the notch filters applied to sentences containing only core modulations (Figure 6C, redness indicates importance for comprehension) gave an overall impression in general agreement with the respective areas of Figure 6A.

It should be noted that, to generate this initial speech MTF, we assumed that spectral and temporal degradations affect the speech signal independently, which allowed us to multiply the normalized comprehension errors. We know, however, from the discrepancy between the comprehension after notch-filtering of core modulations, and the comprehension after notch-filtering of all modulations (namely, removal of intermediate spectral modulations is more detrimental to performance if higher spectral modulations have been removed as well), that there must exist some spectrotemporal interdependence. We also assumed that the MTF is symmetric along positive and negative modulation frequencies, in other words, that the gain in the MTF for joint spectrotemporal modulations corresponding to down-sweeps equals the gain for up-sweeps. Although we have not further explored the interdependence of the spectral and temporal modulations, our joint spectrotemporal modulation filtering technique opens the door to future studies directly assessing the degree of interdependence and potential asymmetry.

The shape of our final speech MTF (temporally band-pass and spectrally low-pass) approximately matches the shape of a psychophysical MTF that was obtained from detection thresholds for broadband moving ripples (corresponding to a single point in the MPS) in white noise [2], but with some important differences. Chi et al. found that the human MTF was low-pass for spectral and temporal modulations, with increases in threshold detection for modulations greater than 2 cycles/octave and 16 Hz (Footnote: Chi et al. state that their MTF is low-pass in the temporal domain but their psychometric function does show that detection at the very low temporal modulations is somewhat more difficult than at the low intermediate temporal modulations). In comparison, if we examined only our low-pass filtering results, we would find modulation cutoff values around 4 cycles/kHz and 12 Hz. (Note that 4 cycles/kHz corresponds to 2 cycles/octave for center frequencies of 500 Hz, and that we too obtained a cutoff value of 2 cycles/octave using log frequency as shown in Figure S1). The estimation of these upper boundaries is therefore very similar between the two studies. However, our complete speech MTF based on the combination of notch and low-pass filters shows a much more restricted area of high gain. For example, while the MTF of Chi et al. is relatively flat all the way to 2 cycles/octave, our speech MTF shows that the lowest spectral modulations (<0.25 cycles/kHz) play a more important role than the higher ones (>0.5 cycles/kHz). There are therefore important differences between the MTF obtained by measuring detections of ripple sounds in noise and the one obtained by performing notch filtering operations on speech. While humans might be equally good at detecting low and intermediate spectral modulations, the lower ones carry more information for speech intelligibility. The intermediate modulations should carry more information for other auditory tasks such as pitch perception.

While animal models of speech perception remain a stretched analogy, models of animal sensitivity to relevant modulations hold more immediate potential. The shape of our speech MTF also resembles the MTFs that have been obtained for mammalian [31] and avian [32] high-level auditory neurons. This correlation between the power of the spectrotemporal modulations in speech (the speech MPS), the MTF resulting from tests of speech intelligibility, the MTF derived from detection of synthetic sounds [2], and the tuning properties of auditory neuron ensembles suggests a match between the speech signal and the receiver. The most informative modulations in speech, and in other animal communication signals, occur in regions of the modulation spectrum where humans show high sensitivity and where animals' high-level auditory neurons have the highest gain [13],[33],[34].

We also examined the role of modulations in the task of recognizing the gender of a speaker. The MPSs of male and female speech differ in the frequency rate at which power is concentrated in the higher spectral modulations (Figure 2). In our MPS representation, the pitch-associated spectral frequencies of male and female speakers showed a bimodal distribution: the two modes correspond to the glottal action of the vocal cords pulsing at ∼150 Hz in adult male speakers and at above 200 Hz in females [22]. The spectral notch filter that removed the high spectral modulation power unique to the female voice confused listeners' percept of gender, such that half of the female stimuli notch filtered between 3–7 cycles/kHz sounded male to subjects. Control stimuli containing only the core modulations, which likewise lack the female-specific modulation power, similarly confused listeners. We conclude that modulations between 3 and 7 cycles/kHz give rise to the percept of female vocal pitch. It is interesting that removal of the modulations underlying the male vocal register did not appear to detract from perception of speaker masculinity. Although fundamental frequencies provide the basis for gender recognition particularly in vowels [35], it has also been shown that the fundamental and the second formant frequency are equally good predictors of speaker gender [36]. Therefore the lower spectral modulations could carry additional gender information, but the acoustic distinction fails to explain the bias for male identification. Alternatively, the perception of vocal masculinity could depend more on gender-specific articulatory behaviors which account for social “dialectal” gender cues distinguishing even pre-pubescent speakers [37].

Our results have implications for speech representation purposes including compression, cochlear design, and speech recognition by machines. In both speech compression applications and signal processing for cochlear design, the redundancy of the speech signal allows a reduction in the bandwidth of a channel through which the signal is represented. For this purpose, limiting spectral resolution has been a favorite solution both because of the robustness of the signal to such deteriorations [6],[29] and because of engineering design constraints for cochlear implants. However, in noisy environments, additional spectral information results in significant speech hearing improvement [20],[25]. Our approach provides a guided solution: after determining the speech MTF, one can selectively reduce the bandwidth of the signal by first representing key spectral modulations and then systematically including the most important adjacent spectrotemporal modulations to capture the greatest overall space within constraints, as illustrated in cartoon form in Figure 6 (see also [2]). Our initial experiment with gender identification, together with research in music perception [38], shows that the most advantageous solution will depend on the task and the desired percept. Finally, the speech MTF could also be used as a template for filtering out broadband noise: a modulation filtering procedure can be used to emphasize the modulations important for speech and to de-emphasize all others. Both the speech compression and the speech filtering operation require a decomposition of the sound in terms of spectrotemporal modulations, as well as a re-synthesis. These are not particularly simple operations (see Materials and Methods), but with advances in signal processing they will become possible in real time. After all, a similar operation appears to happen in real time in the auditory system [12],[21],[39].

## Materials and Methods

### Ethics Statement

Subjects gave written consent as approved by the Committee for the Protection of Human Subjects at University of California, Berkeley.

### Subjects

Native American-English speakers of mixed gender (20 in the low-pass experiment, aged 18–34 yr; and 17 in the notch experiment, age range 18–36 yr) volunteered to participate in the study. Audiograms showed that their hearing thresholds were normal from 30 to 15,000 Hz; one subject was excluded due to high-frequency hearing loss.

### Stimuli Materials

Acoustically clean recordings of spoken sentences were obtained from the soundtrack of the Iowa Audiovisual Speech Perception videotape [40]. The soundtrack was digitized at 32 kHz sampling rate in our laboratory using TDT System II hardware. This corpus consists of 100 short complete sentences read without emotion by six adult male and female American-English speakers. See Figure 1 for the spectrogram of one example, “The radio was playing too loudly.” The corpus has been used by previous studies of speech perception [5],[6]. The original speech sentences were normalized for power. The synthetic degraded speech signals were generated from this original set by a novel filtering procedure performed on the log spectrogram, as described below.

### The Modulation Power Spectrum

The modulation power spectrum (MPS) of a sound is the amplitude spectrum of the 2D Fourier Transform of a time-frequency representation of the sound's pressure waveform [3]. The MPS can be estimated for a single sound (e.g. one sentence) or for an ensemble of sounds (e.g. 50 sentences from adult male speakers). In our analysis, the time-frequency representation is the log amplitude of a spectrogram obtained with Gaussian windows. Gaussian windows are used because of their symmetry in time-frequency and because they result in time-frequency representations that are more easily invertible [41]. As in cepstral analysis [23], the logarithm of the amplitude of the spectrogram is used to disentangle multiplicative spectral or temporal modulations into separate terms. For example, in speech sounds, the spectral modulations that constitute the formants in vowels (timbre) separate from those that constitute the pitch of the voice (Figure 2B). The MPS is then the amplitude squared as a function of the Fourier pairs of the time and frequency axis of the spectrogram of the log amplitude of this spectrographic representation. We call these two axes the temporal modulations (in Hz) and the spectral modulations (in cycles/kHz). One of these two axes must have positive and negative frequency modulations to distinguish upward frequency modulations (e.g., cos(ω_s_f-ω_t_t)) from downward modulations (e.g., cos(ω_f_f+ω_t_t)). By convention, we use positive and negative temporal modulations. The time-frequency resolution scale of the spectrogram (given by the width of the Gaussian window) determines the upper bounds of the temporal and spectral modulation in an inverse relationship as a result of the uncertainty principle or time-frequency tradeoff. The time-frequency scale must therefore be chosen carefully so that modulation frequencies of interest are considered. The choice of time-frequency scale can be made in a somewhat systematic fashion by using a value for which the shape of the modulation spectrum does not change very much. At these values of time-frequency scale, most of the energy in the modulation spectrum would be far from the boundaries determined by the time-frequency tradeoff [3]. For analyzing our original and filtered signals, we used a time-frequency scale given by a Gaussian window of 10 ms in the time domain or 16 Hz in the frequency domain 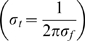. For obtaining the MPS of sound ensembles, sounds in their spectrographic representation were divided into segments of 1 s and the MPS for each segment was estimated before averaging to obtain a power density function. The boundaries of the modulation spectrum at the time-frequency scale of 10 ms–16 Hz are 50 Hz and 31 cyc/kHz. At this time-frequency scale, approximately 90% of the power in the modulation spectrum was found for temporal modulations below 25 Hz and for spectral modulations below 16 cycles/kHz, justifying the choice (Figure 1). Moreover, the temporal and spectral modulation cutoffs correspond approximately to the critical modulation frequency at which amplitude modulated tones and noise start to promote a pitch percept [33]. Thus, when we use this particular time-frequency scale, the temporal modulation frequencies analyzed are perceived predominantly as temporal changes, while higher temporal modulations (those above 50 Hz) which would mediate a percept of pitch are found along the spectral modulation axis. Using wider frequency filters might cause spectral modulation power that is plotted high on the ordinate (e.g., 5 cycles/kHz corresponding to a 200 Hz pitch) to appear instead at a correspondingly high temporal modulation (200 Hz) on the abscissa.

For the modulation filtering operation described below we used other time-frequency scales which were adapted to the filter's cutoff frequencies and thus improved the required spectrogram inversion step in that process.

The MPS can be obtained from a time-frequency decomposition with a linear frequency axis (resulting in spectral modulations in units of cycles/kHz), or from a decomposition with a log frequency axis (resulting in spectral modulation in units of cycles/octave). The log frequency axis is a better model of the decomposition that occurs in the auditory periphery, but we found that the linear-frequency scale is a better decomposition for describing sounds that have harmonic structure. We suggest that higher level neurons may be equally well described as representing either linear or log scale frequency [42]. In any case, both representations are useful. To be able to compare our results to other published work, we additionally obtained the speech MPS and psychometric curves using the log-frequency representation. These results are shown in Figure S1.

### Synthesis of Degraded Speech

The sentences were degraded by a novel modulation filtering procedure. In brief, the sound is first represented in its spectrographic representation using a log-spectrogram calculated with Gaussian windows as described above. Then a new log-spectrogram is obtained by a 2D filtering operation. This filtering operation is performed in the Fourier domain of the modulation amplitude and phase in the following way. First the 2D FFT of the log spectrogram is calculated. Then the amplitudes of specific temporal and spectral modulations that we want to filter out are set to zero. The inverse 2D FFT yields the desired filtered log-spectrogram. After exponentiation, the spectrogram is then inverted using an iterative spectrogram inversion algorithm [43]. We then verified the procedure by calculating the spectrogram and MPS of the filtered sound. For a measure of the errors introduced by spectrogram inversion, we squared the differences between the desired spectrogram and the spectrogram obtained, and divided by the desired spectrogram power, summing the resulting values over time and frequency. Across the 100 stimulus sentences in the control condition, the residual error at the end of 20 algorithm iterations averaged 2.5%. When the 100 sentences were low-pass filtered in one step to create stimuli with only the core modulations, the average residual error after the 20 algorithm iterations was 6.3%. The modulation filtering was written in Matlab using modified code from Malcolm Slaney's Auditory Toolbox for the spectrogram inversion routine [44]. The complete program is available upon demand. The iterative method improves upon earlier overlap-and-add methods that had to compensate for the retention of phase information that unintentionally preserves some spectral information targeted for removal [7],[8].

For the low-pass modulation filtering procedure, the time-frequency scale of the spectrogram was adjusted depending on the desired modulation frequency cutoffs of the modulation filter. For example, if the amplitude of spectral modulation frequencies above 2 cycles/kHz was to be set to zero, then using a time-frequency scale where spectral modulations were represented only up to values approaching 2 cycles/kHz gave better results. In this example, one could use a time-frequency window in the spectrogram of 1.25 ms–128 Hz to obtain a MPS with boundaries at 402 Hz and 3.9 cycles/kHz. Such adjustments made the inverting process much more efficient. Moreover, for low-pass filtering only, one could take this procedure to the extreme and calculate the spectrogram at a time-frequency scale that corresponds exactly to the modulation frequency cut-off of the filter. In that case, the spectrogram would not require any additional filtering and the spectrogram inversion routine can be by-passed altogether. One can instead directly obtain the filtered sounds by using the amplitude envelopes in each frequency band of the spectrogram and using these to modulate a set of narrowband signals of the same bandwidth and center frequency but unitary amplitude. These unit-amplitude narrowband signals can be obtained from Gaussian white-noise that is decomposed through the same spectrographic filter bank [45] or, equivalently, by generating them directly using an analytic signal representation [46]. In the analytical representation the amplitude is set to 1 and the instantaneous phase is random but band limited so that the instantaneous frequency remains within the band. In this study, this direct method was used to generate the low-pass modulation-filtered sentences. The modulation filtering that involved notch or band-stop filtering was done with the complete spectrogram filtering and inverting procedure. In the direct methods, the frequency cutoff for temporal frequencies is inversely related to the frequency cutoff for spectral frequencies but the conjugate boundary was always far from the limits being considered here. For example, a 49 Hz low-pass temporal filter had a conjugate spectral frequency cutoff of 32 cycles/kHz and any temporal filtering with cutoff frequencies below 49 Hz has spectral modulations cutoffs higher than 32 cycles/kHz (Figure 3A and 3B). Because of this relationship the panels C and D of Figure 3 could be merged into one plot that would show a unimodal (inverted U) psychometric curve as a function of a spectrotemporal cutoff (as in Figure S1). More details on these sound synthesis procedures and on time-frequency scale effects can be found in [46] and [3]. A control (unfiltered) speech sentence was obtained by inverting the unfiltered log-spectrogram obtained with the 10 ms–16 Hz time-frequency scale (low-pass experiment) or 5 ms–32 Hz scale (notch experiment). The control sentences sounded very similar to the original sentences and yielded high levels of intelligibility.

Errors calculated during resynthesis depend on the bandwidth of the time-frequency scale. Residual errors in the control case of spectrogram inversion without filtering would barely be affected by changing the time-frequency scale from 5 ms–32 Hz to 1.25 ms–128 Hz (2.92% vs. 2.52% after 20 iterations, averaged over all 100 sentences). Similarly, in the case of temporal and spectral low-pass filtering leaving only core modulations, this time-frequency change would make a minimal improvement in the residual errors (5.49% vs. 6.29%). However, in the case of low-pass spectral modulation filtering with a 2 cycles/kHz cutoff, the 128 Hz time-frequency scale would double residual errors (12.18% vs. 6.41%). Using the 128 Hz time-frequency scale for temporal low-pass filtering with a 6 Hz cutoff would similarly increase residual error (5.64% vs. 2.02%).

### Experimental Procedures

All sounds were presented through headphones (Sennheiser HD265 Linear) to subjects who sat in a sound attenuated chamber. An audiogram from 30 Hz to 15 kHz was obtained initially for each subject, using an adaptive staircase procedure (Tucker Davis Technologies software PsychoSig) and subjects who had thresholds of 20 dB above normal were excluded.

For the comprehension test, the sentences were embedded in Gaussian white noise (0–20 kHz). The white-noise lasted 6 seconds and the sentences (filtered and control) started at random times between 300 ms and 2 s after the onset of the noise. The white noise was played at a level of 65 dB SPL (B&K Level Meter, A-weighting, measured with headphone coupler from B&K). The modulated speech sentences were played at 3 different levels: 72 dB, 67 dB, and 62 dB SPL (B&K level meter, A-weighting, peak level with slow integration, headphone coupler). The 5 dB attenuation steps were obtained using a programmable attenuator (Tucker Davis Technologies). The signal to noise ratios (SNR) calculated from the SPL measurements of the speech and noise signals were therefore +7, +2 and −3 dB. We also calculated the SNR in terms of the RMS values of the sound pressure waveform of the noise and speech and found almost identical values (6.7 dB, 1.7 dB and −3.3 dB). These SNRs were chosen in pilot data to yield complete sigmoidal psychometric tuning curves in the low-pass filtered conditions, and almost perfect speech intelligibility for the control condition [47]. Furthermore, these SNRs cover the 3 dB SNR level that presents little difficulty for normal listeners but reduces comprehension in the hearing impaired [48],[49].

Subjects listened to the sentences at their own pace, pressing a button to elicit the next stimulus. They were instructed to type whatever words they heard followed by whether they perceived the speaker's gender to be male or female. Subjects were asked to guess if necessary, but not to force sentences into making sense if any words did not make sense together. The typed response files were scored for the percentage of words reported correctly, with an algorithm to compensate for small spelling errors. Baseline performance under control conditions and with +2 dB SNR was around 90%.

During an experiment each subject heard all 100 sentences in the corpus without repetitions, so that each sentence was pseudorandomly assigned only to one normal (control) or filtered condition at one level. The SNR levels and the filtering conditions were presented in pseudorandom order. The notch-filtered sentences were presented only at +2 dB SNR.

## Supporting Information

Audio S1 Example sentence under control condition. Mp3 file after conversion from the original wave file of an example stimulus sentence in Figure 1A. No modulation filtering was performed under this condition controlling for spectrogram inversion.
(0.09 MB MPG)

Audio S2 Low-pass modulation filtering at 0.5 cyc/kHz. Mp3 of an example sentence (Figure 3E) with the most extreme spectral modulation filtering (with a low-pass cutoff of 0.5 cyc/kHz).
(0.09 MB MPG)

Audio S3 Low-pass modulation filtering at 3 Hz. Mp3 of the example sentence with the most extreme temporal modulation filtering tested (having a low-pass cutoff of about 3 Hz; Figure 3F).
(0.09 MB MPG)

Audio S4 Low-pass modulation filtering at 4 cyc/kHz. Mp3 of the example sentence with the spectral modulation filtering at which comprehension became significantly worse (cutoff 4 cyc/kHz; Figure 3G).
(0.09 MB MPG)

Audio S5 Low-pass modulation filtering at 12 Hz. Mp3 of example sentence with the temporal modulation filtering at which comprehension became significantly worse (cutoff 12 Hz; Figure 3H).
(0.09 MB MPG)

Audio S6 Example sentence with core modulations. Mp3 of the example sentence containing only the core of essential modulations below 7.75 Hz and 3.75 cyc/kHz (Figure 4G).
(0.09 MB MPG)

Audio S7 Spectral notch filter producing gender misidentification. Mp3 of the example sentence after spectral modulations between 3 and 7 cyc/kHz were filtered out (Figure 4F). Listeners misreported the gender of about half the female speakers.
(0.09 MB MPG)

Figure S1 Modulation power spectrum and performance with linear time-frequency scale. (A) The top panels in the figure show the modulation power spectrum (MPS) of speech (American English) calculated from a time-frequency representation of the sound using a logarithmic frequency filter bank (log-f). The modulation spectrum is shown for male and female speakers. As was the case for the modulation spectrum estimate with a linear frequency filter bank (Figures 1 and 2), the log-f speech modulation spectrum shows a power law distribution of energy and some degree of non-separability between spectral and temporal modulations. However, in the linear modulation spectrum, the spectral modulations in cycles/Hz distribute into clearly separate regions corresponding to pitch and formant energy (Figure 2), whereas in the log-f modulation spectrum the corresponding modulations overlap in a single triangular region below 4 cycles/octave. In addition, in this speech corpus at this time-frequency scale, the harmonic structure of women's vocalic sounds creates a repeated pattern of spectral modulations. The log-f spectrogram was obtained with logarithmically-spaced Gaussian filters with a bandwidth of 0.0138 octaves. (B) The line graph replots on a linear spectral modulation axis the comprehension of sentences after log-f low-pass filtering. The resulting psychometric curve includes low-pass filter cutoffs from 1/4 cycles/octave to 256 cycles/octave, but these can be interpreted as low-pass spectral filtering on the left side of the peak and low-pass temporal filtering on the right side of the peak, as follows. The sound pressure re-synthesis of these sentences used the direct method, where the filtered amplitude was obtained by decomposing the sound into a set of narrowband signals with the frequency bandwidth given by the modulation frequency cutoff, and the filtered phase was obtained from Gaussian white-noise that is decomposed through the same filter bank [40],[41]. For high modulation frequency cutoffs, because of the time-frequency tradeoff, this method effectively low-pass filters the amplitude envelope. In a log frequency representation, the temporal frequency cutoff depends on the center frequency. We show the corresponding temporal cutoff for the frequency band centered at 1 kHz in parentheses under the relevant x-axis labels. The left side of the figure can therefore be compared to the psychometric curve shown in Figure 3C, and the right side to Figure 3D. The left side shows that speech comprehension remains very good with representations having filter bands as wide as 0.25 octaves (the sigma parameter corresponding to 2 cycles/octave cutoff [14]) but that it degrades rapidly with wider frequency bands, particularly in noisy conditions. As in our interpretation of the linear frequency results, this steep decline occurs when spectral modulations that correspond to formants and formant transitions are filtered out. On the right side of the curve, the critical temporal modulation cutoffs are approximately twice as large in this plot as in the linear frequency plot, suggesting that humans cannot easily use the faster temporal information that is present in filters above 500 Hz to compensate for the loss of that information in the lower frequency bands.
(4.37 MB TIF)
