# Supplementary figures and images for: The Modulation Transfer Function for Speech Intelligibility
Source: PLoS Comput Biol. 2009 Mar 6;5(3):e1000302. doi: 10.1371/journal.pcbi.1000302 (PMC2639724; doi:10.1371/journal.pcbi.1000302)

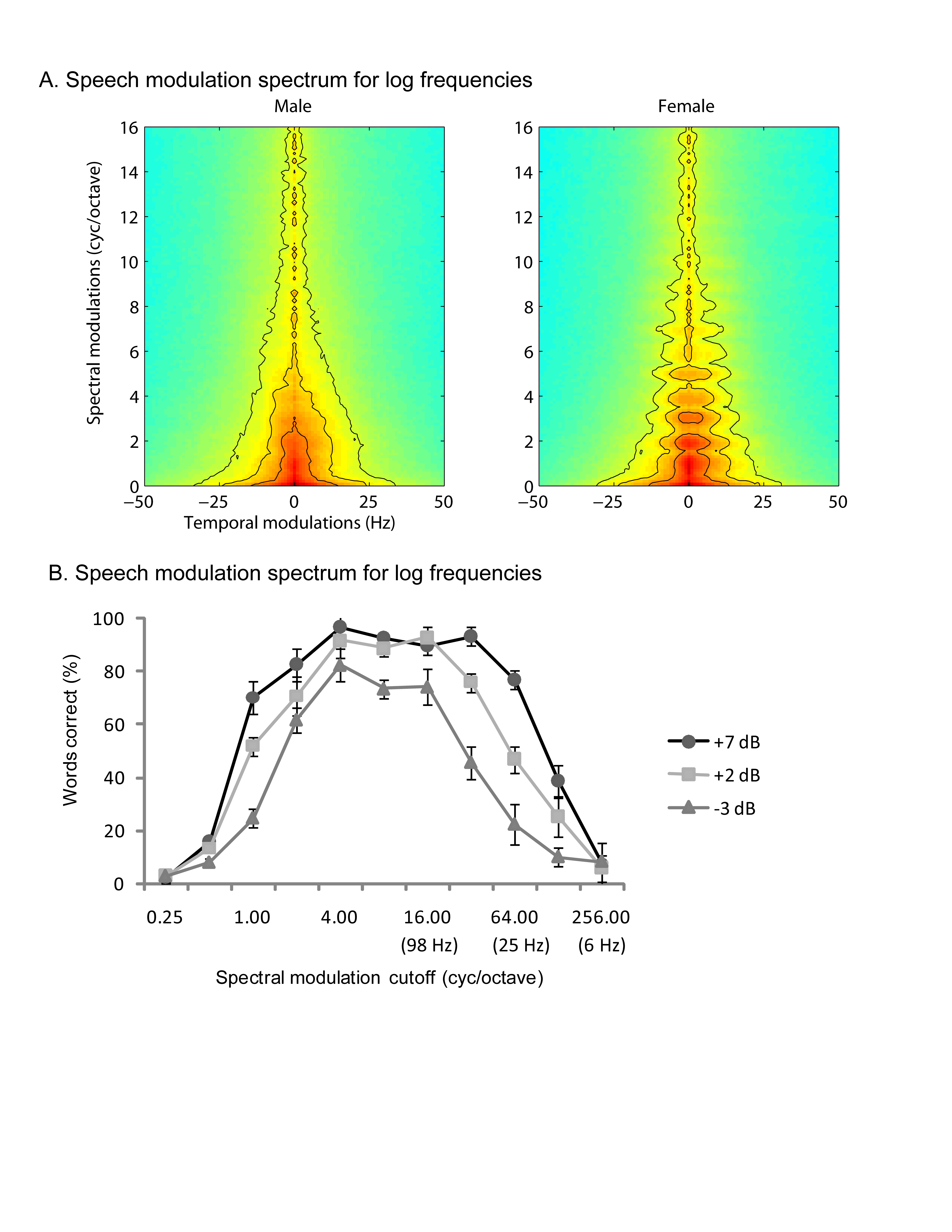

Supplement: Figure S1 — Modulation power spectrum and performance with linear time-frequency scale. (A) The top panels in the figure show the modulation power spectrum (MPS) of speech (American English) calculated from a time-frequency representation of the sound using a logarithmic frequency filter bank (log-f). The modulation spectrum is shown for male and female speakers. As was the case for the modulation spectrum estimate with a linear frequency filter bank (Figures 1 and 2), the log-f speech modulation spectrum shows a power law distribution of energy and some degree of non-separability between spectral and temporal modulations. However, in the linear modulation spectrum, the spectral modulations in cycles/Hz distribute into clearly separate regions corresponding to pitch and formant energy (Figure 2), whereas in the log-f modulation spectrum the corresponding modulations overlap in a single triangular region below 4 cycles/octave. In addition, in this speech corpus at this time-frequency scale, the harmonic structure of women's vocalic sounds creates a repeated pattern of spectral modulations. The log-f spectrogram was obtained with logarithmically-spaced Gaussian filters with a bandwidth of 0.0138 octaves. (B) The line graph replots on a linear spectral modulation axis the comprehension of sentences after log-f low-pass filtering. The resulting psychometric curve includes low-pass filter cutoffs from 1/4 cycles/octave to 256 cycles/octave, but these can be interpreted as low-pass spectral filtering on the left side of the peak and low-pass temporal filtering on the right side of the peak, as follows. The sound pressure re-synthesis of these sentences used the direct method, where the filtered amplitude was obtained by decomposing the sound into a set of narrowband signals with the frequency bandwidth given by the modulation frequency cutoff, and the filtered phase was obtained from Gaussian white-noise that is decomposed through the same filter bank [40],[41]. For high modulation f [file pcbi.1000302.s008.tif]
